# Positional effect of phosphorylation sites 266 and 267 in the cytoplasmic domain of the E2 protein of hepatitis C virus 3a genotype: Interferon Resistance analysis via Sequence Alignment

**DOI:** 10.1186/1743-422X-8-204

**Published:** 2011-05-05

**Authors:** Shazia Rafique, Muhammad Idrees, Muhammad Ilyas, Abrar Hussain, Muhammad Ali, Liaqat Ali, Sadia Butt, Samia Afzal, Irshad Ur Rehman, Sana Saleem

**Affiliations:** 1 Division of Molecular Virology & Molecular Diagnostics, National Centre of Excellence in Molecular Biology, 87-West Canal Bank Road Thokar Niaz Baig Lahore-53700, University of the Punjab Lahore, Pakistan

## Abstract

**Background:**

Interferon is well thought-out as the key defence against all infections including HCV. The only treatment for HCV infection is pegylated interferon alpha (IFN-α) but unluckily more than half of the infected individuals do not act in response to the cure and become chronic HCV carriers. The mechanism how HCV induce interferon resistance is still elusive. It is recently reported that HCV envelope protein 2 interacts with PKR which is the interferon-inducible protein kinase and which in turn blocks the activity of its target molecule called eukaryotic initiation factor elF2. Sequence analysis of Envelope protein reveals it contains a domain homologous to phosphorylation sites of PKR andthe translation initiation factor eIF2alpha. Envelope protein competes for phosphorylation with PKR. Inhibition of kinase activity of PKR is postulated as a mechanism of to interferon (IFN) resistance.

**Results:**

Present study involves the insilico investigation of possible role of potential phosphorylation in envelope 2 protein of 3a genotype in interferon resistance. Envelope protein coding genes were isolated from local HCV isolates, cloned and sequenced. Phylogenetic analysis was done and tertiary structure of envelope gene was predicted. Visualization of phosphorylation in tertiary structure reveals that residue 266 and 267 of envelope gene 2 are surface exposed and their phosphorylation may compete with the phosphorylation of PKR protein and possibly involved in mediating Interferon Resistance.

**Conclusion:**

A hybrid in-silico and wet laboratory approach of motif prediction, evolutionary and structural analysis has pointed out serine 266 and 267 of the HCV E2 gene as a hopeful claimant for the serine phosphorylation. Recognition of these nucleotide variations may assist to propose genotype precise therapy to avoid and resolve HCV infections.

## Background

Hepatitis C virus (HCV) is a major cause of hepatitis which reduces the quality of life of some 170 million people worldwide [1]. HCV infection is frequently associated with chronic liver diseases and development of hepatocellular carcinoma. In Pakistan propensity of HCV infection in local population is ≥6% with prevalent genotype 3a [2]. The patients infected with genotype 3a respond efficiently to interferon therapy but unfortunately the rate of reoccurrence of infection is also very high and after relapse the patients show resistance to interferon therapy. Recent studies showed resistance to interferon in HCV infection has been partially ascribed to functional inhibition of PKR which is interferon induced anti-viral protein [3-7]. HCV is an enveloped sense, single stranded RNA virus belonging to family Flaviviridae. Its genome is 9.6 kb in size and encodes 10 proteins which are synthesized as a poly-protein precursor and subsequently processed by host as well as viral proteases to yield ten mature proteins. HCV also encodes an eleventh protein, ARFP or F that is produced by translational frame shifting from the core region [8]. Viral proteins are expressed in a cap-independent manner by means of an internal ribosome entry site (IRES) located in the 5' UTR [9-11].

The IFN system is the first line of defence against viral infection in mammals [12]. IFNs are glycoproteins commonly known as cytokines which are released by the cells during infections.Activation of type I IFN (IFN-α & β) genes at transcription level is mainly triggered by viral double-stranded RNA present in infected cells [13,14]. Upon infection the virus particles replicate inside the host cell ultimately the cell dies and viral particles are released that can infect surrounding cells. However, the infected cell can warn neighbouring cells of a viral presence by releasing interferon. The neighbouring cells, in response to interferon signalling, produce large amounts of an enzyme known as (PKR). PKR is aserine/threonine kinase found in cells in dormant state.PKR is induced by interferon and activated upon autophosphorylation.It plays an important role in cellular antiviral defence as well as in apoptosis, signal transduction, and transformation [15]. Activation of PKR by autophosphorylation occur upon binding to its regulator, dsRNA molecules [16,17].This permits the enzyme to phosphorylate its substrates. Best known of these is translational initiation factor eIF2, which is phosphorylated on serine 51 of its subunit. Phosphorylation of eIF2a controls a number of cellular processes, most important of which is the blockage of protein synthesis. Phosphorylation of many cellular and viral proteins, including the human immunodeficiency virus transactivator protein, Tat [18,19], and 90-kDa proteins from rabbit reticulocytes [20] and human cells is mediated by PKR [21-24]. The roles of the other phosphorylation events are as yet unknown. Different genotypes of HCV exhibit different rates of response to IFN-alpha and these variants are characterized by mutations that may be accountable for IFN-alpha resistance. Two HCV proteins that have been involved in IFN resistance through inhibition of its downstream protein kinase (PKR) are NS5A and E2 [17,25]. The E2 glycoprotein are supposed to be the first viral components that come in contact with the host cell surface receptors, and elicits production of neutralizing antibodies against the virus, and is involved in viral morphogenesis. It binds to external loop of CD81, a tetraspanin found on the surface of many cell types including hepatocytes [26]. This viral envelope glycoprotein is an obvious candidate for vaccine development as it is chosen target for humoral and cell-mediated immune responses [27,28]. Taylor et al., 1999 reported that envelope protein 2 of HCV contains a 12 amino-acid sequence domain which is homologous to the autophosphorylation site of PKR and initiation factor eIF2a, which is growth they considered it a possible mechanism by which HCV circumvents the antiviral effect of IFN and cause chronic infections and hepatocellular carcinoma. They also reported that E2 proteins with a PePHD sequence identical to genotypes 2 and 3 of HCV, which are known IFN sensitive genotypes, showed only a weak inhibitory effect against PKR activity [28].

## Methods

### PCR amplification of E1E2 genes & cloning

Hepatitis C virus RNA of local 3a genotype was isolated from the serum of a chronic HCV carrier using RNA isolation kit (Gentra, Life Technologies, USA) according to the given kit protocol. The cDNA was synthesized using gene specific anti sense primer and amplified after addition of sense primer. The required amplified product was of approximately 1700 bps. The required band was excised from the gel and the DNA was extracted with DNA isolation kit (Fermentas Inc. Germany). The amplified sequence encoding E1E2 genes were cloned in pcDNA3.1 vector (Invitrogen Tech USA) between Hindi III and EcoR I sites. Transformation: The plasmid was used to transform competent cells. Competent cells that have taken up plasmid wereselected by using 100 g/ml ampicillin and 12.4 ug/ml of tetracycline.

### Colony PCR

To identify bacteria harbouring cloned E1 and E2 genes, individual colonies are used to directly inoculate PCR reactions. The PCR reactions were prepared with 5 pmol each of vector-specific primers T7 (TAATACGACTCACTATAGGG) and BGH Following a single round of amplification; products are isolated on a 1.5%, ethidium bromide-stained agarose gel, a successful cloning reaction being visualized as a product at approximately 2 kb. Colonies identified as possessing a desired clone are then used to inoculate a 3 ml LB culture containing 100 g/ml ampicillin, shaking at 225 rpm overnight at 37°C the plasmids was isolated through plasmid isolation kit by (fermentas). Cultures are used to prepare a plasmid stock Quantification of the plasmid prep is performed on nano drop. Isolated DNA was sequenced. At least three clones were sequenced using in both directions according to the sequencing protocol.

### Sequence analysis

The DNA strands from both PCR products and plasmids were sequenced by using specific sense and anti sense primers the sequences reported in this paper have been deposited in genebank database (Accession no: EU399722). Approximately 350 ng of plasmid DNA used for each sequencing reaction, containing 5 pmol of primer, 2 ul of big dye, 2 ul of sequencing buffer (applied biosystems) in a total volume of 10 ul. Sequencing profile was 94°C for 20 s, 58°C for 20 s 60°C for 4 min, repeated 30 times, transferred to a 0.5 ml microcentrifuge tube, labelled DNA is then precipitated with 50 ul of absolute ethanol. The DNA was kept at room temperature for at least 30 min and centrifuged at 14000 g for 30 min. The supernatant was discarded the DNA pellet washed twice with 250 ul of 70% ethanol, centrifuged at 14000 g for 20 min each time. The pellet was dried and DNA was analyzed using an ABI prism sequencer.

### Phosphorylation sites Prediction

**Figure 1:**
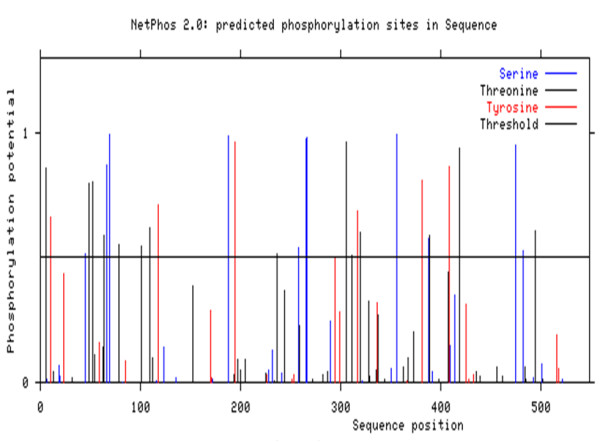
**Phosphorylation sites predicted in the local HCV envelope gene sequence**.

To check post translational modifications in the local 3a sequences of the envelope genes in silico N-linked phosphorylation sites were predicted using the freely available online servers i.e. NetPhos 2.0 developed with neural network which predicts phosphorylation sites in sequences and structure [29] and Scanite which predicts target motifs for different kinases [30]. Scanite predictions were made on the "Low Stringency" to identify as many sites as possible. Further analysis was done using these predicted sites (Figure 1).

### Protein Structure Prediction and Analysis

**Figure 2:**
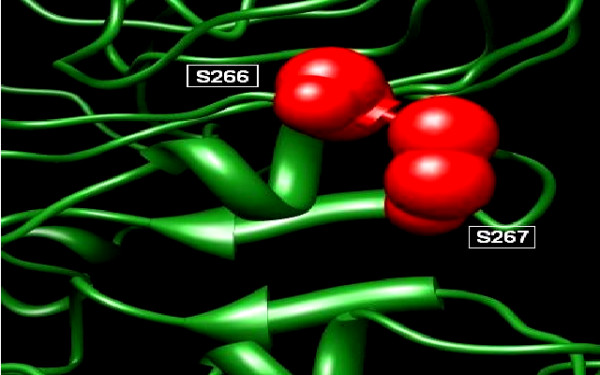
**Visualization phosphorylation sites S266 and S267 in Tertiary structure of the envelope gene**.

Due to the unavailability of template model in Protein Data Bank (PDB) server [31] ab-initio model was developed using I-TASSER [32]. Sequences were uploaded and models were obtained. The phosphorylation sites predicted with two servers were shown in the 3D structure. Chimera [33] and SWISS PDB viewer [34] was used to conclude whether potential phosphorylation sites were surface opened or masked. Five residues were observed on the exposed surface of the model (aa: S66, S266, S267, S356, T311). The most reliable sites are visualized in the (Figure 2).

**Figure 3:**
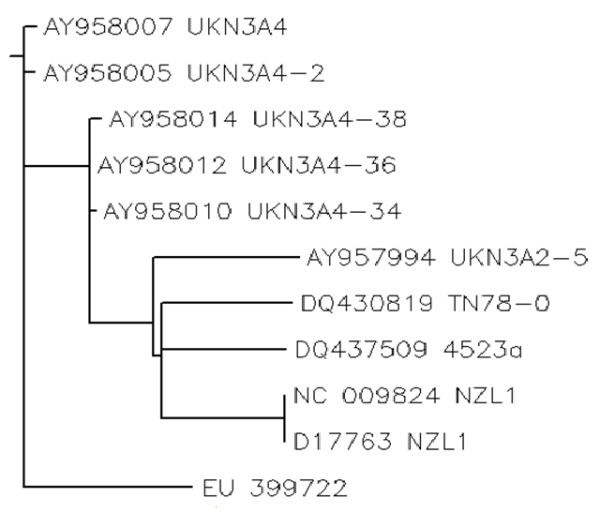
**Phylogeny of local envelope gene sequence with other reported sequences for 3a genotypes from different regions of the world**.

Phylogenetic Analysis: Protein sequences of local envelope gene sequence and other reported sequences for 3a genotypes from different countries of the world (Japan, China, USA, UK) were retrieved from NCBI. All sequences (EU399722, NC009824, D17763, AY958007, AY958005, AY958014, AY958012, AY958010, AY957994, DQ430819 and DQ437509) were then aligned by using CLUSTALW [35]. A neighbour joining tree was constructed using PHYLIP [36] (Figure 3).

## Results

To characterize the role of envelope genes in disease progression we cloned the amplified PCR product in mammalian expression vector pc DNA 3.1. This vector has a CMV promoter which represents an effective mean to transduce eukaryotic cells for transient and stable expression studies. The cloned genes were sequenced in both direction and the consensus sequence was made and submitted to NCBI GeneBank database. The assigned Accession number to this sequence is EU399722. Local envelope 2 gene sequence was compared with other reported sequences for 3a genotypes from different regions of the world to find out the percentage nucleotide identity (PNI) (Table 1). Multiple sequence alignment phylogenetic study was done by using CLUSTALW[35] (Figure 3).

**Table 1:** Comparison between local and reported envelope gene sequences.

ACCESSION No	GENOTYPE	COUNTRY	IDENTITIES
EU 399722	3a	PAKISTAN	100%
NC_009824 NZL1	3a	JAPAN	1484/1632(90%)
D17763 NZL1	3a	JAPAN	1484/1632(90%)
AY958007 UKN3A4	3a	UK	1475/1631(90%)
AY958005 UKN3A4-2	3a	UK	1472/1631(90%)
AY958014 UKN3A4-38	3a	UK	1470/1631(89%)
AY958012 UKN3A4-36	3a	UK	1470/1631(88%)
AY958010 UKN3A4-34	3a	UK	1469/1631(88%)
AY957994 UKN3A2-5	3a	UK	1472/1631(88%)
DQ430819 TN78-0	3a	USA	1452/1632(87%)
DQ437509 4523a	3a	CHINA	1454/1631(87%)

Potential phosphorylation sites in the cytoplasmic domain of the E2 protein was predicted, which may be possibly involved in mediating interferon resistance. Phosphorylation site predictor has the tendency to over-predict, therefore stringency was increased and only those motifs were selected that received a NetPhos score of 0.8 or higher or predicted by both NetPhos [29] (Figure 1) and Scansite [30]. The final yield was 31 putative sites (Ser- 11, Thr- 14 and Tyr- 6). Fine of them were predicted by both the servers.

The phosphorylaton sites must be present on the surface of the protein. To find this the local sequence was analysed for finding the surface accessibility by using online server NetSurfP [37] (Table 2). The putative sites were then visualized in the 3D protein structure using SWISS PDB Viewer[34] (Figure 2).

**Table 2:** Summary of predicted tyrosine phosphorylation sites.

**Site**^**a**^	**aa**^**b**^	**Context**^**c**^	***NetPhos***^***d***^	***Scansite***^***e***^	**NetSurfP**^**f**^	**I-TASSER**^**g**^
66	*S*	ATTASVRSH	0.869		E	Yes
266	*S*	PRRLSSCKP	0.978	Y	E	Yes
267	*S*	RRLSSCKPI	0.983	Y	E	Yes
356	*S*	LRPPSGRWF	0.992		E	No
311	*T*	VKAATVCGP	0.512		E	No

a) Phosphorylation sites in the local 3a sequences of the envelope genes.

b) S indicates Serine; T is for Threonine and Y for Tyrosine predictions.

c) Region where the phosphorylation sites are available.

d) Predicted sites by NetPhos with a score of ≥ 0.8. Dashes indicate lack of Phosphorylation sites in that position.

e) Y indicates predicted sites in sequence on "Low Stringency". Dashes indicate lack of Phosphorylation sites in that position.

f) E indicates Exposed sites and B indicates Buried sites. Surface accessibility calculated by NetSurfP.

g) Ab-initio 3D model was constructed by using I-TASSER server.

The PDB structure was developed by using Ab-initio protein structure predictor server I- TASSER[32]. From all this analysis two sites were found to be most reliable phosphorylation sites i.e. S266 and S267. Scansite was used for finding the phosphorylation interaction motifs and found that S66, S266 and S267 interact with the PKC, Casein Kinase, SRC Kinase and AKT Kinase which were then investigated in GeneCards and was confirmed from UniGene and UniProt[38] (Table 3).

**Table 3:** Interacting enzymes predicted by Scansite.

**Site**^**a**^	**Enzyme**^**b**^	Gene Card	**UniGene**^**c**^	**UniProt**^**c**^	Full Name
S66	PKC alpha/beta/gamma	PRKCA	Yes	P17252	Protein kinase C, alpha
	Casein Kinase 1	CSNK1G2	Yes	P78368	casein kinase 1, Gamma 2
Y226	Src Kinase	SRC	Yes	P12931	V-Src sarcoma (Schmidt-Ruppin A-2) viral oncogene homolog (avian)
	Protein Kinase A	PRKACG	Yes	P22612	Protein kinase, cAMP-dependent, catalytic, gamma
S267	PKC alpha/beta/gamma	PRKCA	Yes	P17252	Protein kinase C, alpha
	Akt Kinase	AKT1	Yes	P31749	v-akt murine thymoma viral oncogene homolog 1

a) Phosphorylation sites in the envelope gene.

b) Observed enzymes by the Scansite.

c) Gene's availability on UniGene and UniProt.

## Discussion

The envelope protein 2 (E2) of hepatitis C virus (HCV) is recently reported to interact with double stranded RNA-dependent protein kinase (PKR) [9,12,15,16,23,25]. HCV envelope protein E2 contains a domain homologous to phosphorylation sites of the interferon-inducible protein kinase PKR and the translation initiation factor eIF2 alpha, which is a target of PKR [23]. As a result E2 competes and inhibited the kinase activity of PKR and blocked its inhibitory effect on protein synthesis and cell growth [18,21,29,32,39]. We aim to find out the role of phosphorylation of envelope protein 2 on interferon & PKR response. Envelope protein coding genes were isolated from local HCV isolates and cloned in mammalian expression vector for further analysis. The cloned fragment was sequenced and the sequence was submitted to NCBI (EU 399722). The local envelope gene sequence was compared with other reported sequences for 3a genotypes from different regions of the world to find out the percentage nucleotide identity (PNI) as variations in the envelope genes accounts for different degrees of disease progression in HCV RNA positive individuals with different genotypes [3,12,25]. In silico N-linked phosphorylation sites were predicted (Figure 1) using the freely available online servers i.e. NetPhos 2.0 developed with neural network which predicts phosphorylation sites in sequences and structure [29] and Scanite which predicts target motifs for different kinases [30]. Scanite predictions were made on the "Low Stringency" to identify as many sites as possible. Further analysis was done using these predicted sites. The final yield was 31 putative sites (Ser- 11, Thr- 14 and Tyr- 6). Fine of them were predicted by both the servers (Table 2). The phosphorylation sites must be present on the surface of the protein. To find this local envelope protein sequence was analysed for finding the surface accessibility by using online server NetSurfP. Due to the unavailability of template model in Protein Data Bank (PDB) server [40], ab-initio model was developed using I-TASSER [32]. Sequences were uploaded and models were obtained. The phosphorylation sites predicted with two servers were shown in the 3D structure. Chimera [33] and SWISS PDB viewer [34] was used to infer whether potential phosphorylation sites were surface exposed or buried in which five residues were observed on the exposed surface of the model (aa: S66, S266, S267, S356, T311). The most reliable sites are visualized in the (Figure 2). From all this analysis two sites were found to be most reliable phosphorylation sites i.e. S266 and S267. Scansite was used for finding the phosphorylation interaction motifs and found that S66, S266 and S267 interact with the PKC, Casein Kinase, SRC Kinase and AKT Kinase which were then investigated in GeneCards and was confirmed from UniGene and UniProt. Protein sequences of local envelope gene sequence and other reported sequences for 3a genotypes from different countries of the world (Japan, China, USA, UK) were retrieved from NCBI. All sequences (EU399722, NC009824, D17763, AY958007, AY958005, AY958014, AY958012, AY958010, AY957994, DQ430819 and DQ437509) were then aligned by using CLUSTALW [35]. A neighbour joining tree was constructed using PHYLIP [41] (Figure 3). IFN normally acts by upregulating expression of many antiviral genes, including the double stranded RNA activated protein kinase (PKR), which in turn inhibits protein synthesis by phosphorylation of the translation initiation factor eLF2. HCV have evolved the strategies to surmount the antiviral effects of PKR [39] and phosphorylation of E2 protein may accounts for IFN resistance. This work will help to identify factors that favour a successful innate immune response against HCV infections.

## Competing interests

The authors declare that they have no competing interests.

## Authors' contributions

MI conceived the study and critically reviewed the manuscript. SR performed, sequenced and analyzed the results. SR, MI and AH drafted the manuscript. MA, LI, SB, SA, IR, and SS participated in data analysis. All the authors studied and approved the final manuscript.
